# Cereals and Cereal-Based Foods: Nutritional, Phytochemical Characterization and Processing Technologies

**DOI:** 10.3390/foods14071234

**Published:** 2025-04-01

**Authors:** Grazia Maria Borrelli, Donatella Bianca Maria Ficco

**Affiliations:** Consiglio per la Ricerca in Agricoltura e l’Analisi dell’Economia Agraria—Centro di Ricerca Cerealicolturae Colture Industriali, 71122 Foggia, Italy

Cereals have historically been recognized as an important part of the human diet. They include a variety of grains, such as wheat, durum wheat, maize, oats, rice, rye, barley, and sorghum, representing two-thirds of human food consumption. Cereal-based foods can be cooked and eaten as whole grains, ground into flour to make a variety of products like bread, pasta, and noodles, or used as ready-to-eat breakfast cereals and snacks [1].

With cereals being an important source of energy and compounds with nutritional and healthy properties, the development of cereal food products with enhanced features presupposes the exploitation of genetic materials with good levels of endogenous nutritional and phytochemical compounds.

This Special Issue comprises a collection of eleven peer-reviewed articles focused on the study of the nutritional/functional and physico-chemical features of raw materials for the improvement of derived cereal-based foods. In total, 75 authors contributed to this collection, which consists of ten research articles and one review article that addresses the most recent advances on these topics. The articles published in this Special Issue covered the characterization of the nutritional and technological properties of cereals to be used for the production of targeted foods (five articles), the study of primary and secondary processing (four articles), and the potential reuse of by-products as new ingredients in formulations for cereal food development (two articles).

Some cereals play an important role in the nutrition of subjects affected by particular pathologies, such as gluten-related disorders or diabetes. Gluten-related disorders, particularly celiac disease, are triggered by the ingestion of gluten proteins naturally present in grains such as rye, wheat, and barley. Patients suffering from celiac disease have a reduced quality of life due to the need to adopt a strictly gluten-free diet, with all the negative nutritional implications due to the characteristics of traditional gluten-free products that are often rich in lipids and poor in vitamins, antioxidants, and fibers [2]. To meet their dietary needs, much research is aimed at investigating other gluten-free cereal species to identify genotypes with good baking performance and good acceptability and technological quality for developing novel and healthier gluten-free products. Among these, sorghum, oat, and maize represent valuable gluten-free raw materials for the development of products with improved nutritional value.

Sorghum is an excellent source of proteins, carbohydrates (starch), vitamins, minerals (such as iron and potassium), other bioactive compounds (especially phenolic and flavonoid compounds), oligo-elements (mainly magnesium and manganese), and fatty acids (ω-6 and 3) [3,4]. Moreover, it has a low glycemic index, making sorghum-derived products beneficial to diabetics, in addition to for people affected by celiac disease or gluten intolerances [4]. Oat is rich in substances beneficial to the human body, including vitamin E (tocols), phenolic compounds, β-glucans, and insoluble fiber [5]. Maize is a good source of starch, proteins, and lipids, and of several bioactive compounds. Different types of maize are characterized by grain color, varying from white to yellow, pink, red, blue or black, and these pigmented maizes are richer in many secondary metabolites, such as phenolic compounds, anthocyanins, carotenoids, and tocols [6]. The nutritional and technological traits of sorghum hybrids, hulled/naked oat varieties, and white/pigmented maize genotypes and landraces were analyzed by Gazza et al. (Contribution 1) to identify species and cultivars expressing better characteristics intended for various processed foods. Differences in the content of protein, total and resistant starch, amylose, and the total dietary fiber, particularly β-glucan, as well as in the content of phenolic compounds and antioxidant capacities were found in the three species. Although all the three species possess technological properties suitable for food processing, their pasting properties, related to the structural changes affecting the starch granules during gelatinization and retrogradation, provided more precise information regarding their aptitude for making particular products. In fact, some oat genotypes showed characteristics suitable for formulations of gluten-free pasta or bakery products, while some maize and sorghum genotypes could be adapted for baking goods. Moreover, other sorghum genotypes were found to be more appropriate for food products that require stable thickening after heating and cooling, like in sauce production.

Among the minor cereals, sorghum is the most investigated, as it can grow in semi-arid conditions, as well as having nutritional peculiarities. This led to much research investigating sorghum genotype variability. Pontieri et al. (Contribution 2) assessed the nutritional and bioactive components, including proteins, carbohydrates, dietary fiber, unsaturated fatty acids, and minerals, of nine inbred varieties grown under typical Mediterranean conditions with the aim of identifying varieties with superior nutritional attributes to utilize in breeding programs.

However, sorghum’s nutritional components, particularly its proteins and minerals, are less available for humans and animals due to presence of anti-nutritional factors, like tannins and phytic acid. In fact, tannins interact with proteins, creating complexes that inhibit protein/enzyme and starch digestibility. Moreover, tannins, similarly to phytate, can also bind to minerals, thus reducing their bioavailability. Therefore, various approaches such as fermentation, germination, and hydrolysis are employed to cope with these limits. Among these, germination represents a cost-effective and efficient technology that facilitates structural modifications of bound phenolic compounds into free forms, improving the nutritional value, generating various bioactive compounds, and also reducing the anti-nutritional factors (tannins, phytates, and protease inhibitors) [7,8]. Kaisoglu et al. (Contribution 3) demonstrated the effectiveness of germination over a period of 7 days in increasing the nutritional and functional qualities of red and white sorghum grains, currently limited to animal feed use, in comparison to native sorghum. They found increased protein, lipid, fiber, minerals and lignin content. The authors also observed a decrease in starch content and modifications to the molecular structure of starch granules in both sorghum varieties that could pave the way for their possible use in human nutrition.

Besides being a source of food for celiacs, maize can have many other fields of food industry application related to the production of Resistant Starch type 5 (RS5). RS5 is highly interesting in the food industry for its versatility and functional properties, having characteristics of non-toxicity, biodegradability, and an ability to selectively release bioactive compounds in the gastrointestinal tract [9]. Consequently, it is important to explore cost-effective and efficient methods to produce it. The formation of RS5, primarily associated with amylose–lipid complexes, is typical of starches with a high amylose content due to their affinity for lipid compounds [10]. However, the parameters governing gelatinization are critical for the successful formation of RS5 [11]. Recently, some studies have also investigated the potential of amylopectin-rich starches to form amylopectin–lipid complexes (ALCs), broadening RS5 sources. Starches rich in amylopectin may be a more accessible source for RS5 formation, as the size of the chains obtained by amylopectin debranching significantly affects their ability to interact with lipids. The type of fatty acid, influencing the formation of complexes with starch molecules, also affects the content of RS5, the enzymatic hydrolysis rate, and the thermal properties of the starch. Specifically, complexes formed between oleic acid and amylopectin-rich (waxy) starch under certain conditions lead to a higher content of RS5 [12]. In this context, Castro-Campos et al. (Contribution 4) analyze the potential of waxy maize starch, which is rich in amylopectin, to develop amylopectin–lipid complexes, with oleic acid (10% *w*/*w*) under different thermal and mechanical conditions. They identify boiling conditions with 45 min of stirring as the optimal conditions for the formation of stable ALCs that, restricting the access of enzymes, showed the highest resistance to the digestion process.

Overall, to obtain superior foods with high nutritional and health value, a decisive contribution derives from the degree of refinement of flours. Cereal wholemeals provide many important nutrients and bioactive compounds with an important role in reducing the risk of developing various chronic diseases and age-related conditions [13]. Dragičević et al. (Contribution 5) evaluated the content of important macro- (protein) and micro-nutrients (mineral elements), as well as bioactive compounds, such as soluble and insoluble functional dietary fiber prebiotic dietary fiber (e.g., cellulose, arabinoxylan, β-glucan, xyloglucan, and fructan) and some antioxidants (glutathione, yellow pigment, and the specific profile of phenolic compounds), in wholemeals of cereals, such as barley, durum wheat, bread wheat, ancient wheats, such as spelt and emmer wheat, and minor crops, such as triticale, sorghum, oat, and rye. The results confirmed the importance of these meals as nutraceuticals. The unique chemical profile of oat and barley reiterates their utility in the production of functional foods. Regarding wheats, some of these compounds are mostly concentrated in ancient genotypes. Despite the lower yields, compared to the modern wheat genotypes, their value is associated with their sustainability, as they are better adapted to low-input and organic agriculture, growing on marginal soils and in dry conditions [14,15].

The second part of this Editorial is mainly devoted to evaluating the effect of processing on the nutritional and technological quality of cereal-based foods. Final quality results from the interaction among many different factors including species, genotype, environment, and primary (milling) and secondary processing steps are presented. For instance, in wheat, milling has an important effect beyond reducing nutritional compounds [16], also altering the structure and functionality of starch granules, thus producing damaged starch (DS) [17]. The content of DS influences the physicochemical and rheological properties of the flours, directly affecting their industrial performance [18]. The degree of starch damage during milling is influenced by the composition of the starch (amylose/amylopectin ratio), the hardness of the grain, and the milling conditions, increasing with the severity of the milling process. Damaged starch granules increased their water absorption capacity during dough preparation, affecting dough mixing and the rheological properties of flour. Furthermore, DS granules are more accessible to endogenous β-amylase hydrolysis, which results in the production of maltose and dextrin [18]. In conventional baking procedures, maltose is used by yeast to ferment to carbon dioxide, causing loaves to rise. An understanding of how the DS content of flour affects the performance of the product has been a very important requirement in the baking industry. Teobaldi et al. (Contribution 6) assessed the morphology of wheat starch granules with different DS, correlating it with the rheological and thermal behavior of starch-based systems. They demonstrated that higher levels of DS determine a decrease in the driving force for water attachment to the starch granules allowing to a weaker water–starch polymer chain interactions and to a reduction in the evaporation temperature that could affect bread quality.

It is also interesting to account for the effect of milling on wheat protein in an industrial setting. In fact, milling also affects protein content in the kernel. Wheat is the main source of protein worldwide. Within the starchy endosperm, protein content and composition have a positive gradient from the center of the endosperm towards the aleurone layer [19,20]. The extent of this gradient highly depends on the cultivar and the level of nitrogen fertilization (N-fertilization) [20]. Milling separates the starchy endosperm from the aleurone layer and bran and grinds this into white flour. It was reported that the last break (BFs) and reduction fractions (RFs) are higher in protein content compared to the other flour fractions due to aleurone and bran contamination [21]. Other than the protein content, the relative gluten content and composition also play a crucial role in determining the functionality of flour. Hermans et al. [22] showed that the sub-aleurone has a significantly higher relative gluten content than the inner endosperm without significant differences in the HMW-GS protein proportion. Hermans et al. (Contribution 7) studied how the protein gradient within the starchy endosperm is reflected in the gluten content and composition of mill fractions, using three cultivars grown without and with N-fertilization (300 kg N ha^−1^). The increasing protein content in successive break fractions (BFs) was shown to reflect the protein gradient within the starchy endosperm. They found an increase in protein content, also in the reduction fractions (RFs), that could be ascribable to increased levels of aleurone and bran contamination. The BFs were used to investigate the magnitude of the gradient in gluten content within the starchy endosperm and assess the effect of cultivar and N-fertilization level on it. They showed that, in all cultivars, the gluten content increased from the inner to outer endosperm to a greater extent without N-fertilization than with it. Furthermore, no consistent gradient for the gluten composition was observed when N-fertilization was applied. This study gives information for the milling industry to manage flour streams of different raw materials and optimize the process.

Secondary processing is used to transform grain/flour into edible products. This process can be studied both to identify the optimal experimental conditions to improve the performance of the end products and to evaluate the effect of the process as such on some qualitative characteristics. These two aspects have been investigated on some puffed cereals and pasta, respectively.

Puffed cereals are ready-to-eat cereal grain formulations suitable for human consumption without requiring further cooking. Non-optimal storage conditions of temperature and moisture could cause microbial spoilage and deterioration affecting their shelf life. Previous studies examined the moisture sorption and plasticity characteristics of biscuits [23] and wafers [24] and the relationship between moisture sorption and the texture of different baked products, such as corn cakes [25], by exploring their glass transition points as a critical factor involved in food deterioration and shelf life. Takahashi and Fujii (Contribution 8) analyzed the rheological and processing properties of puffed brown rice, barley, adlay, and amaranth by assessing alterations in the breaking behavior, monomolecular adsorption, moisture content, and glass transition points to evaluate the effects of moisture absorption on their rupture properties. All puffed cereals were stored under different temperatures and humidity conditions. They identified the ideal conditions for ensuring the desired crispy texture by maintaining a humidity content of <8% and a storage temperature of <40 °C.

In the case of pasta products, color plays an important role in attracting consumers and the pasta market. Troccoli et al. (Contribution 9) analyzed the traits involved in color expression, such as carotenoid pigments, yellow and brown indices, and oxidative enzymes such as lipoxygenase, responsible for pigment loss that occurs during pasta-making [26,27], and peroxidase and polyphenoloxidase, which influence brown hue, negatively affecting yellowness [28]. This study was carried out in semolina and pasta produced from eighteen durum wheat genotypes grown in eight environments. After processing, a decrease in the yellowness, measured both as the carotenoid pigment content and the yellow index, was observed, with a slight increase in the brown index. A multiple regression analysis performed on semolina traits demonstrated that the semolina yellow index was the main predictor for pasta color, while enzyme activities did not emerge as having a determining role. As a significant effect of genotype, environment, and their interaction was demonstrated on all traits investigated, to identify the genotype/environment that better combine all traits that positively (carotenoid pigment content and yellow index) or negatively (oxidative enzyme activities and brown index) influence color expression, the High-Performance Index tool, developed by Troccoli et al. [29], was applied. This information is very useful to predict final pasta color and perform suitable durum wheat breeding programs for its improvement.

With growing demand for functional, natural, and low-calorie ingredients, extensive research is being carried out to identify novel components that can be useful in the enrichment of formulations for various cereal-based products. Functional ingredients may also be derived from by-products or food processing waste, with the aim of sustainably using natural resources, providing additional economic benefits to food businesses fitting into a circular economy context.

Among the different by-products, mango peel is interesting as it is rich in dietary fiber, polyphenols, proteins and carotenoids, making it a promising source of functional ingredients in the production of various processed foods, such as fermented beverages, jellies, chips, pasta, biscuits, bread, and snacks [30]. Aviles et al. (Contribution 10) developed nutritious, high-quality snacks made from oats, dehydrated mango pulp, and mango peel flour. The authors prepared ten different mixtures, each with unique combinations and proportions of the three components. The optimal formulation able to produce the best snack quality consisted of 44.38% oats, 5.36% mango peel, and 29.24% mango pulp. Furthermore, they found that the low water activity (Aw) values enhanced quality stability at room temperature, ensuring a longer shelf life. These snacks were found to be microbiologically safe, easy to handle, visually appealing, and capable of maintaining their shape during market distribution.

Another example of by-product use is represented by rice bran [31]. Due to its unique fatty acid composition and functional components, the derived oil is recommended by the World Health Organization as one of the three major healthy edible oils, along with corn and sesame oils. It is rich in tocopherol, γ-glutamate, phytosterol, and other unsaponifiable substances, which have remarkable antioxidant, anti-inflammatory, cancer-preventive, hypoglycemic, lipid-lowering, and neuroprotective effects, becoming more beneficial to human health [32]. Rice bran is produced in large quantities annually. However, due to its low cost and high susceptibility to spoilage (3–5 days), it is mainly used to feed animals, while only 2–5% is used as a raw material for the production of rice bran oil for high production costs [32]. Huang et al. (Contribution 11) investigated the nutritional value and safety of rice bran oil. Furthermore, they have considered the stability of raw rice bran materials and the extraction and refining processes of rice bran oil and discussed food applications and sub-health regulations. The authors stated that a delayed stabilization treatment of rice bran seriously affects the overall quality of rice bran oil. Compared with traditional solvent extraction, new extraction technologies have been reported that have improved the yield and nutritional value of rice bran oil, but most of them are still in the basic research phase due to a lack of economical and applicable supporting production equipment. Therefore, despite the functional value and the health benefits of rice bran oil, its extraction is difficult to transfer to industry, thus limiting its production and consumption.

The articles published in this Special Issue range from the characterization and valorization of genetic material, from a food-chain perspective, to obtaining nutritionally superior cereal-based products suitable for target consumers in a sustainability perspective.
